# Is greater milk production associated with dairy cows who have a greater probability of ruminating while lying down?

**DOI:** 10.3168/jdsc.2021-0159

**Published:** 2021-11-25

**Authors:** C.J. McWilliams, A.J. Schwanke, T.J. DeVries

**Affiliations:** Department of Animal Biosciences, University of Guelph, Guelph, ON, N1G 2W1, Canada

## Abstract

The objective of this study was to determine whether associations exist between position while ruminating (lying vs. standing) and milk and component production in dairy cows. Data from 30 lactating Holstein cows were assembled from 2 studies in which cows were milked by automated milking system (AMS) and fed a partial mixed ration (PMR) in feed bins that recorded intake behavior. Rumination and lying behavior were monitored using automated neck- and leg-based sensors, respectively. Each cow was monitored over 2 separate 2-wk treatment periods. To estimate position while ruminating for each 2-h period of the day for each cow, a conditional probability was calculated to determine the probability that any rumination time and lying time were occurring at the same time in any 2-h period. These probabilities (RwL), and all behavioral data, were summarized per cow per 2-h interval, and then averaged per day and per 2-wk period, along with milk yield and component data. Cows averaged (mean ± standard deviation) 1.9 ± 1.1 lactations and 85.5 ± 55.2 d in milk, and weighed 668.5 ± 96.0 kg. Data included rumination time (557.7 ± 41.1 min/d), lying time (703.9 ± 65.3 min/d), idle standing time (520.1 ± 83.2 min/d), PMR feeding time (204.7 ± 48.5 min/d), PMR dry matter intake (DMI; 21.8 ± 4.6 kg/d), AMS pellet provision (4.6 ± 1.6 kg/d), total DMI (26.4 ± 4.5 kg/d), milk yield (42.4 ± 7.2 kg/d), milk fat content (3.75 ± 0.51%), and milk protein content (3.21 ± 0.32%). Greater rumination time and lying time were associated with greater RwL probability (mean = 0.19 ± 0.02; range = 0.14 to 0.23). The RwL probability tended to be positively associated with total DMI and milk fat content, was associated with milk protein content, but was not associated with any measures of milk yield. The results indicate that in a free-traffic AMS, cows who have greater probability of ruminating while lying down spend more time ruminating and lying, and tend to consume more total dry matter and produce milk with greater components.

## Highlights

• Greater lying time and rumination time were associated with greater probability of ruminating while lying down.
• Probability of ruminating while lying down was predictive of milk fat content, milk protein content, and DMI.
• This research highlights the importance of promoting greater lying time for lactating dairy cows.

## Graphical Abstract

**Figure:**
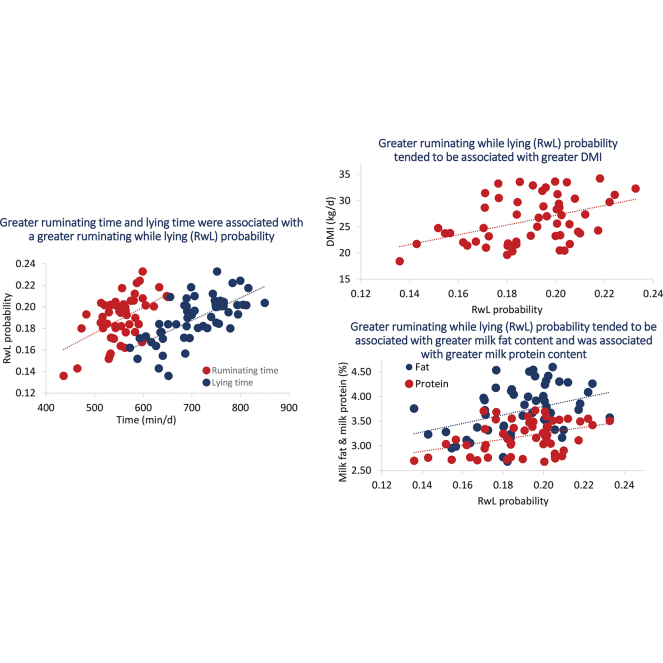
**Summary:** The objective of this study was to determine whether an association exists between the probability of position while ruminating (lying vs. standing) and production in dairy cows. The results demonstrate that a greater probability that cows are ruminating while lying is positively associated with rumination time, lying time, total DMI, milk fat percent, and milk protein percent.

The process of rumination allows dairy cows to digest forages, specifically the plant cell wall components, which are largely unavailable to nonruminant species. Rumination is one of the most pronounced behaviors that cows express, to which they dedicate 9 to 12 h/d on average (Aikman et al., 2008; Beauchemin, 2018). Rumination is essential for the reduction of feed particle size, which not only increases surface area to allow for microbial colonization and degradation (Aikman et al., 2008; Antanaitis et al., 2018; Beauchemin, 2018; Li et al., 2020), but also allows feed to pass through the reticulo-omasal orifice (Aikman et al., 2008), thus reducing the gut-fill effect and promoting further feed intake (Antanaitis et al., 2018). In turn, greater feed intake, and the resultant nutrients obtained from digestion, are positively associated with milk yield (Johnston and DeVries, 2018). Thus, associations of rumination time with DMI and milk yield are predicted. In support of that, when controlling for parity and BW, Johnston and DeVries (2018) observed that DMI tended to be positively associated with rumination time. Further, positive relationships have been reported between milk production and rumination time (Johnston and DeVries, 2018; Antanaitis et al., 2019). Specifically, Johnston and DeVries (2018) demonstrated, in high-producing Holstein cows, that for every 1-h increase in rumination time, milk yield was predicted to increase by 1.26 kg/d.

Rumination behavior may also be associated with the lying behavior of dairy cows. Schirmann et al. (2012) reported a positive association between rumination and lying time with 2-h periods of the day, indicating that periods of rumination are more frequent when cows are lying down. Rumination follows a diurnal pattern, with more rumination occurring at nighttime (Antanaitis et al., 2019), when cows are more often lying down (Schirmann et al., 2012). Acatincăi et al. (2010) reported that cows ruminate 63.4% of the time in a lying position and 36.5% of the time in a standing position, whereas Phillips and Leaver (1986) reported that cows will spend 1 h/d ruminating while standing and 6 h/d ruminating while lying. It is interesting to note that if lying time is restricted, rumination time is reduced, but not to the same extent because cows will compensate by ruminating while standing (Tucker et al., 2021). The biological consequences of posture during rumination (lying or standing) have not been established, despite the prior suggestion that this needs to be researched (Krawczel et al., 2012).

Thus, the objective of this study was to determine if there is an association of position while ruminating (i.e., standing vs. lying) with milk and component production responses in lactating dairy cows. Specifically, we aimed to associate (1) behavioral predictors with the probability that any rumination time and lying time were occurring at the same time, and (2) production outcomes with the probability that rumination time and lying time were occurring at the same time. We hypothesized that a greater probability of time spent ruminating while lying down would be associated with greater rumination time, feed intake, and milk production.

A database containing behavior, DMI, and production data for 30 lactating Holstein dairy cows, with 28 d of data per cow (collected in two 14-d study observation periods) was assembled from 2 studies (Schwanke et al., 2019, 2020) conducted at the University of Guelph Elora Research Station – Ontario Dairy Research Center (Guelph, ON, Canada). In both studies, cows (n = 15 per study) were kept in a single freestall pen (with access to 30 stalls, having a mattress base and bedded with chopped straw) where they were individually assigned and trained to eat from automated feed bins (Insentec B.V.) that contained a partial mixed ration (**PMR**) fed 1× daily. Cows were milked in an automated milking system (**AMS**; DeLaval, Tetra Laval Group), with permission to enter the AMS when 4 h had elapsed since the previous milking and predicted milk yield for that milking exceeded 9.0 kg, with additional grain offered in the AMS (amount varying depending on experimental treatment). Average parity, DIM, and BW of the study cows are reported in Table 1. The use of cows in each study was approved by the University of Guelph's Animal Care Committee; all experimental procedures complied with the guidelines of the Canadian Council on Animal Care (2009).

**Table 1:** Descriptive statistics (mean, SD, minimum, maximum) of cow information, intake, behavior, and production variables included in the models (n = 60 cow-period observations^1^)

Item	Mean	SD	Minimum	Maximum
Cow data and intake				
RwL probability^2^	0.19	0.02	0.14	0.23
Parity^3^	1.9^3^	1.12	1	4
DIM^4^	85.5	55.2	28	190
BW, kg	668	96	533	907
PMR^5^ DMI, kg/d	21.8	4.62	13.9	31.1
AMS^6^ pellet allocation, kg/d	4.6	1.61	2.73	6.70
Total DMI, kg/d	26.4	4.47	18.4	34.2
DMI as % of BW	4.0	0.43	2.89	4.98
Behavior				
PMR feeding time, min/d	205	49	99	313
PMR feeding rate, kg of DMI/min	0.1	0.04	0.05	0.22
Rumination time, min/d	558	41	436	6,500
Rumination time, min/kg of DMI	22	3	15	27
Standing time, min/d	731	65	590	868
Lying time, min/d	709	65	572	850
Milking time, min/d	22	6	12	36
Idle standing time, min/d	503	78	316	670
Milking frequency, no./d	3.6	1.00	2.00	5.79
Milk production				
Milk yield, kg/d				
Milk	42.4	7.16	29.0	61.5
4% FCM	37.6	7.48	22.9	58.9
ECM	40.3	8.19	24.4	64.5
Milk composition, %				
Fat	3.7	0.51	2.68	4.60
Protein	3.2	0.32	2.68	3.72
Milk component yield, kg/d				
Fat	1.6	0.39	0.86	2.70
Protein	1.4	0.31	0.79	2.13
Log_10_SCC, log_10_ cells/mL	4.5	0.40	3.98	5.96

1 Data were collected across two 14-d treatment periods per cow (6 d/cow for milk component data) and averaged to create values for each 14-d treatment period.

2 RwL probability = conditional probability (potentially ranging from 0 to 1) calculated as the probability that any rumination time and lying time within each 2-h time period per day were occurring at the same time. Probabilities across 2-h time periods per day were averaged per day per cow, and then by 14-d treatment period.

3 Parity distribution was 1 (n = 32 cow-periods), 2 (n = 10 cow-periods), 3 (n = 10 cow-periods), and 4 (n = 8 cow-periods); parity was categorized as 1 (primiparous) and 2+ (multiparous).

4 DIM = mean days in milk of cows at the beginning of each 14-d data collection period (cow-period).

5 Partial mixed ration.

6 Automatic milking system.

Treatments in each study were implemented in a crossover design, with 2 dietary treatments over two 28-d periods. The first 14 d served as an adaptation period, followed by 14 d (15 to 28) of data collection (Schwanke et al., 2019, 2020). Treatment diets for the first study consisted of a less nutrient dense PMR with 6.0 kg DM/d of concentrate in the AMS, and the other treatment was a more nutrient dense PMR with 3.0 kg DM/d of concentrate in the AMS. On a DM basis, the base PMR ingredients were 27.5% corn silage, 26.7% alfalfa haylage, 10.3% high-moisture corn, and 10.3% PMR supplement; The diet major nutrients averaged 16.7% CP, 21.6% ADF, 32.7% NDF, and 1.66 Mcal/kg NE_L_ (Schwanke et al., 2019). The other study treatment diets consisted of the same base PMR with treatments offering either 6.0 or 3.0 kg of DM/d at the AMS (Schwanke et al., 2020). On a DM basis, the major ingredients of the base PMR were 34.9% corn silage, 35.0% alfalfa haylage, 14.6% high-moisture corn, and 13.5% PMR supplement. The diet major nutrients averaged 18.0% CP, 20.4% ADF, 30.9% NDF, and 1.68 Mcal/kg NE_L_ (Schwanke et al., 2020).

Behavior data, as summarized across cows and studies in Table 1, were collected similarly in both source studies. Partial mixed ration feeding behavior and DMI were monitored continuously throughout each study using the automated feed bins (Insentec B.V.). As validated by Chapinal et al. (2007), the bins recorded feed intake (kg) and feeding time (min) for each individual animal for each visit to the feed bin. These data were summarized into 2-h intervals and then summarized by day, and finally by period. The AMS pellet delivered (kg as-fed) was recorded from the AMS system, as it allocated and recorded pellet offerings per visit to the AMS, which was then summarized by day (DeLaval). Standing and lying behavior data were recorded with electronic data loggers (HOBO Pendant G Data Logger, Onset Computer Corp.), as validated by Ledgerwood et al. (2010), that were attached to one of the rear legs of the cows. Measurements were taken using leg orientation at 1-min intervals. From these data, time spent lying down and standing (min/d) were calculated. Idle standing time, defined as the time the cows were standing while not ruminating, eating, or milking, was calculated as standing time (min/d) – feeding time (min/d) – time spent in the AMS (min/d). Rumination activity was monitored using an electronic data logger (HR-TAG-LD, SCR Engineers Ltd.), as validated by Schirmann et al. (2009), that was attached to the neck collar of each cow. Data from this system provided cumulative rumination time (min) for each 2-h time block throughout each day.

Given that rumination data were limited to being recorded in 2-h time blocks, to align the lying behavior data with that rumination data, the lying data were summarized by cow into 2-h time blocks per day that coincided with the rumination data time blocks. To estimate position while ruminating, for each 2-h period of the day for each cow, a probability (ranging from 0 to 1) was calculated to determine the probability that any rumination time and lying time were occurring simultaneously (**RwL probability**). The RwL probability was calculated by dividing rumination time (min/2 h) by 120 and multiplying that by lying time (min/2 h) divided by 120:

$$RwL=\left(\frac{Ruminating\;time}{120}\right)\times \left(\frac{Lying\;time}{120}\right).$$

Following these calculations, the RwL probabilities were then averaged per day per cow.

Production data collected in these studies included milking frequency, milking time, milk yield, milk composition, and milk component yield. Milk yield, milking frequency, and duration of milking were recorded by the AMS using DelPro 4.5 (DeLaval). In both studies, milk samples were collected from each cow from each milking (through a 24-h period) on 3 d per treatment period (d 20, 24, and 28; Schwanke et al., 2019, 2020). These samples were sent to a DHI testing laboratory (Lactanet, Guelph, ON, Canada) for component analysis (fat, CP, and SCC) using a Fourier transform infrared full-spectrum analyzer (Milkoscan FT+ and Milkoscan 6000; Foss). Production data were summarized by study period (14 d) where all variables were represented as average daily values over the 14-d period (Schwanke et al., 2019, 2020). Fat-corrected milk was calculated by correcting milk yield for 4% milk fat composition: FCM = [0.4 × milk yield (kg/d)] + [15.0 × fat yield (kg/d)] (NRC, 2001). Energy-corrected milk was determined as ECM (kg/d) = (0.327 × kg of milk) + (12.95 × kg of fat) + (7.2 × kg of protein) (Tyrrell and Reid, 1965).

Within each study, cow BW was measured by bringing cows to a scale (1–20W scale, Ohaus) on 2 consecutive days at the start of the adaptation period, the start of the data collection period, and the end of each data collection period.

Cows were individually exposed to both treatments within the studies from which their data were sourced. For the current analyses, the experimental unit was the cow-period observation, each of which was the average of daily data (behavior, production, and RwL probability) collected for each cow during 14-d data collection periods, per treatment, in each respective study. Data were averaged per treatment period to improve the accuracy of the estimate of the true mean for each predictor and outcome variable. Before analyses, all data were screened for normality using the UNIVARIATE procedure of SAS 9.4 (SAS Institute Inc., 2013). The SCC data were right-skewed; thus, to achieve normality, the log_10_ value was calculated and used for analyses.

Mixed multivariable linear regression models were built to assess whether there were associations between behavioral measures (Table 1) as predictors and RwL probability as an outcome, as well as between RwL probability as the predictor of production outcomes (Table 1), with cow-level predictors (parity, DIM, BW, DMI as % of BW, milking frequency; Table 1) tested as covariates. Models were constructed using the MIXED procedure of SAS. Multiple observations per cow within each source study (i.e., different treatments) were accounted for in each model by including treatment in the repeated statement. Thus, the random effects were study and cow within treatment and study (subject of the repeated statement). Compound symmetry was selected as the covariance structure on the basis of best fit according to Schwarz's Bayesian information criterion. Degrees of freedom for fixed effects were estimated using the Kenward-Roger option in the MODEL statement.

All predictor variables hypothesized to be related to the outcome of interest were initially screened for unconditional associations in univariable analyses. Any predictor variables that were liberally associated with the outcome (*P* < 0.25) were considered for inclusion in the final model. Spearman correlation coefficients were calculated for all predictor variables that were considered for inclusion in the multivariable model to detect issues of collinearity. Consequently, if the correlation coefficient was greater than |0.6|, then either the variable that made the most biological sense was used, or that with the lower *P*-value. A confounding variable was defined as a non-intervening variable whose removal resulted in a >25% change in the coefficients of significant variables in the final model. Predictor variables in each model were checked for interaction by including all biologically appropriate 2-way interaction terms into the model. Variables were retained in the final multivariable model if they were significant at *P* ≤ 0.05 or tendencies at 0.05 < *P* ≤ 0.10, if they acted as a confounder, or were part of a significant interaction term.

To our knowledge, this project created the first estimation of ruminating position by creating an RwL probability, as reported in Table 1. A positive association of RwL probability and rumination time was observed (Table 2), whereby those cows with higher rumination times were more likely to be doing that while lying down. Rumination activity occurs during rest periods, typically at night, but also throughout the day (Schirmann et al., 2012). Further, there is some evidence in the literature that cows will ruminate more while lying than while standing; Acatincăi et al. (2010) and Phillips and Leaver (1986) both reported that cows will spend a greater proportion of their time ruminating while lying than ruminating while standing. In an observational study of freestall-housed, parlor-milked cows by Lindgren (2009), it was observed that outside of the 3 h/d dedicated to milking, cows were lying, standing, ruminating while lying, and ruminating while standing for 318.6, 264.9, 330.6, and 185.4 min/21 h, respectively. They determined that cows spent 35.9% of their time ruminating while standing. Modifying the RwL calculation created in the current study to estimate the RwL probability in the Lindgren (2009) study, on an average daily basis, for cows in that study, it was determined that their RwL probability was 0.21. This furthers the validation of the RwL probability that was created for the current study, which found an RwL probability of 0.19, and demonstrates that the values obtained herein are reasonable.

**Table 2:** Final multivariable linear regression model for factors associated with probability that cows were lying down and ruminating at the same time (n = 60 cow-periods^1^)

Variable	RwL probability^2^		
	β^3^	SE	*P*-value
Intercept	−0.017	0.0157	<0.001
Rumination, min/d	0.00033	0.000019	<0.001
Lying time, min/d	0.00025	0.000013	<0.001

1 Data were collected across two 14-d treatment periods per cow and averaged to create values for each 14-d treatment period per cow.

2 RwL probability = conditional probability calculated as the probability that any rumination time and lying time within each 2-h time period per day were occurring at the same time. Probabilities across 2-h time periods per day were averaged per day per cow, and then by 14-d treatment period (average ranging from 0.14 to 0.23).

3 β = estimated regression coefficient.

The RwL probability in the current study was also positively associated with lying time (Table 2), indicating that cows with greater lying duration had a greater probability of ruminating while lying down. In our model of RwL probability, we were not able to include idle standing time, because that measure was negatively associated with lying time (r = −0.78; *P* < 0.001). However, on its own, RwL probability was negatively associated with idle standing time (r = −0.57; *P* < 0.001), indicating that those cows with greater average inactive standing time and lesser lying time had a lower probability of ruminating while lying down. As noted by Tucker et al. (2021), there is conflicting evidence for the relationship between ruminating and lying in the literature; Schirmann et al. (2012) reported no correlation, whereas Stone et al. (2017) observed a negative correlation. There is significant evidence in the literature that rumination follows a diurnal pattern, with more rumination occurring at night (DeVries et al., 2009; Schirmann et al., 2012) when cows are typically lying down. Additionally, Beauchemin (1991) noted a common belief that rumination time and lying time are associated. The conflicting evidence in regard to the relationship between ruminating and lying may be related to the fact that cows can also ruminate while standing (Schirmann et al., 2012). Interestingly, and in support of the current findings, Cooper et al. (2007) restricted lying time in cows and observed that while cows will ruminate while standing, there was a numerical decrease in overall rumination time.

Dry matter intake (kg/d) tended to be positively associated with RwL when controlling for BW (Table 3), indicating that cows who had a greater probability of ruminating while lying had higher DMI. Greater DMI associated with greater rumination has been documented in the literature; Johnston and DeVries (2018) reported that for every 1-h increase in rumination time per day, DMI is predicted to increase by 0.96 kg/d; meanwhile, Clément et al. (2014) observed an increase of 0.0031 kg/d DMI for every 1-h increase in rumination time. Alternatively, Schirmann et al. (2012) did not observe any correlations between DMI, rumination time, and lying time when observing dry cows. To our knowledge, our finding is the first reported association of DMI with position while ruminating. This positive association may be due to an increased rate of passage by cows who are more likely to ruminate while lying. As fermentation activity passes its peak, the specific gravity of feed particles increases and the particles sink within the rumen, thus passing through the reticulo-omasal orifice (Aikman et al., 2008). This process may be more effective when cows are lying because their hind end is likely at a lower angle than their front end, furthering the specific gravity of feedstuffs, increasing the rate of passage, and increasing DMI. Alternatively, it is possible that salivation rate, which varies with activity (Beauchemin, 2018), is greater when cows are lying while ruminating, and this in turn may affect rumen particle turnover and ultimately DMI. These hypotheses, however, are both speculative and need to be tested in future work.

**Table 3:** Final multivariable linear regression models for factors associated with total DMI, milk fat content, and milk protein content (n = 60 cow-periods^1^)

Variable	Total DMI (kg/d)			Milk fat (%)			Milk protein (%)		
	β^2^	SE	*P*-value	β	SE	*P*-value	β	SE	*P*-value
Intercept	−7.01	3.56	0.054	0.64	0.707	0.37	2.05	0.375	<0.001
RwL probability^3^	32.4	17.8	0.075	5.77	3.02	0.06	6.01	1.97	0.004
BW, kg	0.041	0.00435	<0.001	—	—	—	—	—	—
DMI as % of BW	—	—	—	0.50	0.153	0.002	—	—	—

1 Data were collected across two 14-d treatment periods per cow and averaged to create values for each 14-d treatment period per cow.

2 β = estimated regression coefficient.

3 RwL probability = conditional probability calculated as the probability that any rumination time and lying time within each 2-h time period per day were occurring at the same time. Probabilities across 2-h time periods per day were averaged per day per cow, and then by 14-d treatment period (average ranging from 0.14 to 0.23).

Milk fat content (%) tended to be positively associated with RwL probability when controlling for DMI as a percent of BW (Table 3), indicating that cows who had a greater probability of ruminating while lying down produced milk of greater fat content. Interestingly, on a univariable level, milk fat yield (kg/d) also tended to be associated with RwL probability (r = 0.25; *P* = 0.06). There is conflicting evidence in the literature on associations of rumination with milk fat. Johnston and DeVries (2018) observed that milk fat content was quadratically associated with rumination time. In contrast, in a study conducted by Andreen et al. (2020) on 2 commercial dairy farms, it was observed on one farm that rumination time and milk fat yield were negatively correlated, whereas on the other farm no relationship was observed. Although this and other previous work has documented an association of rumination with milk fat percent (Grant et al., 1990; Li et al., 2020), to our knowledge, the present study is the first to report that position while ruminating may be associated with milk fat percent. We hypothesize that this association exists because cows who spend less time ruminating while lying spend less time chewing, resulting in reduced saliva buffering and lower ruminal pH, increasing the risk of milk fat depression. More work is needed to test this hypothesis.

Similar to milk fat content, milk protein content (%) was positively associated with RwL probability (Table 3), indicating that cows who had a greater probability of ruminating while lying down produced milk of greater protein content. On a univariable level, milk protein yield (kg/d) also tended to be associated with RwL probability (r = 0.23; *P* = 0.08). Interestingly, Johnston and DeVries (2018) observed that milk protein content was negatively associated with rumination time, but milk protein yield was positively associated with rumination time. Andreen et al. (2020) observed that rumination time and milk protein percentage were negatively correlated for both farms in their trial. Although rumination time and milk protein percent have been associated in the past, the current study is the first to associate position while ruminating with milk protein percent. Caccamo et al. (2014) observed that increased chewing activity and greater rumen pH were associated with increased milk protein percent. It could be hypothesized that a decrease in time spent ruminating while lying will lead to decreased chewing activity and, thus, decreased saliva production and lower rumen pH. Thus, the associations observed in the current study between increased milk protein content and increased RwL probability are potentially caused by the greater rumination performed by cows who are lying down more.

Contrary to our prediction, no measures of milk yield were associated with RwL probability. A lack of evidence regarding position while ruminating and production outcomes has been noted in the literature (Krawczel et al., 2012; Tucker et al., 2021). This result was surprising, because the literature would suggest that greater rumination time (which was associated with RwL probability in the present study) is associated with both DMI (Clément et al., 2014; Johnston and DeVries, 2018) and milk yield (Soriani et al., 2012; Johnston and DeVries, 2018; Antanaitis et al., 2019). Other factors that affect milk production include ration composition (Beauchemin, 2018) and health status of the cow (Soriani et al., 2012). Thus, these and other factors may have influenced milk yield more in our study cows than their position while ruminating. The current results and those from other studies may indicate that total rumination time is more important than position while ruminating for total milk yield, whereas position while ruminating may be important more for milk component concentration.

A limitation that may have prevented even more significant findings from this study was the fact that we could not calculate the actual proportion of rumination time that occurred while lying down. Although the positional sensors attached to the rear leg of each cow recorded minute-by-minute observations of lying time, the rumination monitors only recorded data in 2-h time blocks. As a result, we were limited to only calculating the probability that any rumination and lying were occurring simultaneously during each 2-h time block. Ideally, in future studies, minute-by-minute observations for both rumination and lying behavior could be made to corroborate the associations detected in the present study. Another limitation that may have affected the findings of this present study is that the data were sourced from 2 previous studies in which different treatment diets were fed. Although dietary treatment was controlled for in our statistical model, that variation in diets between treatments and studies could have affected both rumination and lying behavior and, thus, the observed associations we detected.

In conclusion, we identified associations between RwL probability and production outcomes in dairy cows. Lying time and rumination time were positively associated with RwL probability. Greater RwL probability tended to be associated with greater DMI and greater milk fat content, and was also associated with greater milk protein content. These results suggest that encouraging lying while ruminating has positive benefits on dairy cow productivity.
